# Co-evolution of Immune Response in Multiple Myeloma: Implications for Immune Prevention

**DOI:** 10.3389/fimmu.2021.632564

**Published:** 2021-02-26

**Authors:** Samuel S. McCachren, Kavita M. Dhodapkar, Madhav V. Dhodapkar

**Affiliations:** ^1^Department of Hematology and Oncology, Emory University School of Medicine, Atlanta, GA, United States; ^2^The Wallace H. Coulter Department of Biomedical Engineering, Georgia Institute of Technology and Emory University, Atlanta, GA, United States; ^3^Aflac Cancer and Blood Disorders Center, Children's Healthcare of Atlanta, Emory University, Atlanta, GA, United States; ^4^Winship Cancer Institute, Atlanta, GA, United States

**Keywords:** myeloma and other plasma cell dyscrasias, immune response, immune checkpoint, MGUS, prevention

## Abstract

Multiple myeloma (MM), a malignant neoplasm of plasma cells that reside in the bone marrow (BM), is universally preceded by a precursor state termed monoclonal gammopathy of undetermined significance (MGUS). Many individuals with MGUS never progress to MM or progress over many years. Therefore, MGUS provides a unique opportunity to surveil changes in the BM tumor microenvironment throughout disease progression. It is increasingly appreciated that MGUS cells carry many of the genetic changes found in MM. Prior studies have also shown that MGUS cells can be recognized by the immune system, leading to early changes in the BM immune environment compared to that of healthy individuals, including alterations in both innate and adaptive immunity. Progression to clinical MM is associated with attrition of T cells with stem memory-like features and instead accumulation of T cells with more terminally differentiated features. Recent clinical studies have suggested that early application of immune-modulatory drugs, which are known to activate both innate and adaptive immunity, can delay the progression to clinical MM. Understanding the biology of how the immune response and tumors coevolve over time is needed to develop novel immune-based approaches to achieve durable and effective prevention of clinical malignancy.

## Introduction

Multiple myeloma (MM) is a malignant neoplasm characterized by progressive growth of clonally transformed plasma cells in the bone marrow (BM), leading to organ dysfunction manifesting as anemia, lytic bone disease, hypercalcemia, and renal failure (1). It is now well-appreciated that nearly all cases of MM are preceded by the precursor phases termed as monoclonal gammopathy of undetermined significance (MGUS) and smoldering myeloma (SMM) (2). In contrast to MM, the precursor states are characterized by a clinically stable biomass of transformed cells that, by definition, does not lead to overt organ dysfunction. However, a proportion of these patients, estimated at 1% per year in the case of MGUS and 10% per year in the case of SMM, do transform to clinical MM requiring therapy. It has been suggested that the precursor phase may originate as early as the third decade of life (3). Therefore, a great majority of the life history of the malignant clone is spent in its precursor stage. Understanding the early events and the mechanisms underlying the pathogenesis of MGUS and the factors that regulate the transition of MGUS to MM is critical to develop rational approaches to prevent clinical cancer.

The concept that clinical malignancy originates in the context of a more common precursor state is not unique to MM but also occurs across nearly all human malignancies. Hematologic malignancies such as MM and their precursor states do however represent useful models to gain basic insights into these early events in cancer biology and immunology, as the precursor lesions cannot be resected and, therefore, are typically monitored longitudinally in patients. In cancer biology, progressive growth and transformation of the precursor states to clinical malignancy resemble evolution of the species, bearing several similarities to Darwinian evolution (4). In this review, we will argue that this evolution is impacted by and occurs in the context of host response, and the coevolution of these two adaptive biologic systems (tumors and immune system) determines the final trajectory of evolution of MM tumors. Recent studies have given a new understanding of how genetics, BM niche, and the immune microenvironment may play distinct, interconnected roles in disease evolution from MGUS to MM. However, it is still unclear which events are causal for disease progression and which events result due to progression. A deeper understanding of the interactions between these factors and myelomagenesis will inform more effective strategies to delay disease progression and target malignant plasma cells.

## Genetic Heterogeneity and Evolution in Myeloma

Analysis of tumor genomes has demonstrated that MM is characterized by a high degree of genetic complexity and clonal heterogeneity. It has been proposed that the development of the malignant plasma cell population occurs as a result of the progressive accumulation of genetic changes (5). Patterns of genomic evolution of MM have been recently characterized (6). Common initiating mutations that confer an initial clonal advantage and disrupt normal plasma cell activity include hyperdiploidy, translocation of immunoglobulin heavy chain, and 13q deletion (7, 8). After these initiating events, increasing genetic alterations, such as copy number variants, insertions/deletions, translocations, aneuploidy, and non-synonymous single nucleotide variations (NS-SNVs), as well as structural genomic alterations have been observed over the progression of the malignant plasma cell populations (9, 10). However, these mutations do not appear in a purely linear fashion. Rather, they occur in a branching manner, leading to a variety of distinct subclones within the tumor population; therefore, the intraclonal heterogeneity model appears to best describe the mutational landscape of MM. These subclones differentially proliferate based on fitness, leading to a diverse and evolving tumor (11). Patients with MM demonstrate an average of five detectable major subclones, further emphasizing the nature of MM as a heterogeneous disease (8, 12, 13). Epigenetic changes in tumor cells also appear over the course of MM (14). Both global hypomethylation and gene-specific hypermethylation appear with a high degree of variability in methylation among patients with MM (15). Deregulation of histone-modifying proteins and chromatin modifiers has been observed in MM, but the significance of downstream modifications to chromatin is undetermined (14, 16–18).

The mechanisms underlying the genomic instability in MM are under active evaluation but involve cytidine deaminases, such as activation-induced cytidine deaminase (AID) and apolipoprotein B mRNA editing enzyme, catalytic polypeptide-like (APOBEC) (19, 20). Mutational signatures of these genes have been detected in MM genomes. Expression of AID in MM cells is impacted by their interaction with dendritic cells (DCs) in a receptor activator of nuclear factor kappa-B ligand (RANKL)-dependent manner, suggesting that genomic instability in tumor cells may be more directly linked to interactions with the tumor microenvironment (20).

Interestingly, the genomes of MGUS and MM clonal plasma cells are rather similar; the majority of the genomic changes seen in MM are already present in MGUS before transformation to full malignancy (13, 21–23). The common initiating mutations of MM previously mentioned, such as IgH translocations and hyperdiploidy, are also already present in MGUS, and all major genetic subtypes of MM are indeed represented in the MGUS stage (7, 9, 10, 24). In this review, we argue that the evolution from MGUS to MM is not solely influenced by the genetic evolution of the tumor but rather by the interactions between the tumor and the BM microenvironment, especially the immune cells.

## Immune Recognition and Surveillance in MGUS and MM

### Evidence for Immune Surveillance in MM

The concept that the immune system mediates surveillance of early tumors has been demonstrated in several models for both spontaneous as well as carcinogen-induced models (25). Effects of the immune system on early tumors have been characterized in three distinct stages: elimination, equilibrium, and escape. Evidence for immune surveillance in MM has emerged both from the murine models and from studies on patients with early lesions. In the Vk*MYC model of myeloma, the immune system provides surveillance against the growth of tumor cells in a CD226-dependent manner (26). The CD226-dependent response was mediated by both NK and CD8+ T cells in a perforin and interferon gamma-dependent manner. The concept of tumor-extrinsic control on the growth of MGUS is supported by studies in humanized mouse models. In a study evaluating the engraftment of MGUS and MM patient bone marrow transplanted into MIS^(*KI*)^TRG6 mice, MGUS tumor cells exhibited progressive growth, suggesting that the dormant nature of the MGUS cells in patients is regulated in part by tumor-extrinsic controls, including the immune system (27). In a preclinical model of syngeneic stem cell transplant for MM, it was observed that TIGIT immune checkpoint blockade led to the prevention of CD8+ T cell exhaustion and successful immune control of MM, supporting the role of immune system in tumor control (28). These studies are also supported by immune recognition of MGUS lesions and the finding that the nature of immune response against MGUS correlates with the risk of progression to clinical MM (29). Clonal T cell expansions have also been observed to be associated with lower tumor burden and survival advantage in patients with MM, supporting a role for the immune system in MM control (30–32).

### Immune Recognition of Human MGUS and MM

As with many cancers, substantial evidence exists indicating immune recognition and activation in the context of MM. Specific humoral and cytotoxic responses to cancer-associated antigens occur in the BM of patients with MM. In MM expressing the cancer/testis antigen NY-ESO-1, specific antibodies and antigen-specific T cells were observed, and these T cells maintained the ability to kill primary MM cells (33). T cells from patients with MM exhibit robust anti-tumor responses when activated by DCs loaded with tumor antigen (34). Natural killer (NK) cell activation and cytotoxicity have also been implicated in anti-MM immunity, with patient-derived NK cells being able to recognize the MM cells (35). An increased number of infiltrating immune cells, including NK cells and T cells, have been observed in the BM of MM (36). MM cells expressing MICA activate γΔ T cells and promote anti-MM cytotoxicity (37). Furthermore, T cells in MM exhibit significant signs of exhaustion, suggesting chronic exposure to target antigen on the MM cells (38). Therefore, the immune system recognizes MM, resulting in activated anti-tumor immunity and tumor-specific immune cells. However, this immune response appears insufficient to fully contain the tumor.

Immune recognition of the tumor does not solely occur after its malignant transformation to symptomatic MM. Rather, studies have demonstrated earlier immune recognition of MGUS cells in the BM. Tumor-specific CD4+ and CD8+ T cell effector responses have been observed in the BM of MGUS (34). T-cell immune responses specific to several cancer/testis antigens were also observed in the BM of MGUS (39). Interestingly, the targets of T-cell response in MGUS appear to be enriched for antigens typically expressed on stem cells. For example, robust humoral and cellular anti-SOX2 immune responses have also been observed in patients with MGUS (40). MGUS T cells have exhibited responses that are specific to an MGUS-associated antigen OFD1 (41). In a prospective clinical trial, the presence of T cells against SOX2 was associated with a reduced risk of progression to clinical MM (29). In the sections below, we will discuss changes in innate as well as adaptive immunity in patients with MM and MGUS.

### Alterations in Innate Immunity

NK cells have been observed at increased frequencies in the BM of both MGUS and MM relative to healthy donors (42–44). However, there are clear functional differences between the NK cells in MGUS and MM. MGUS NK cells generally maintain their capacity for activation and antibody-dependent cellular toxicity, while MM NK cells exhibit marked functional deficits (45, 46). NK cells appear to lose tumor lysis ability correlating with the clinical stage in MM (47). The NK cells in the BM of MM exhibit decreased expression of activating NK receptors, including NKG2D, NCR3, and CD244 (42, 48). The NK cells in MM have also been observed to have increased PD-1 expression, indicating a potential mechanism for tumor immune evasion, as well as a potential therapeutic target (49). These data indicate a role for increased populations of active NK cells early in the course of disease in containing the malignant plasma cells. Subsequent loss of this early anti-tumor NK cell activity seen in MGUS may allow the progression to symptomatic MM. MM cells also express killer immunoglobulin-like receptor (KIR) ligands that interfere with NK cell cytotoxicity. Anti-KIR antibodies have been observed to enhance MM cell lysis *in vitro* in combination with daratumumab and daratumumab plus lenalidomide (50). Early clinical trials of an anti-KIR antibody in combination with the immunomodulatory agent lenalidomide, an approved MM treatment that is known to expand and activate NK cells, have proven to be safe (51).

Natural killer T (NKT) cells have also been observed to have deficits in MM, including defective interferon-γ production and decreased frequencies. NKT cells from MGUS patients have preserved interferon-γ production relative to MM patients (52, 53). Lenalidomide enhances NKT cell cytokine production and proliferation *in vitro* (54). Another study assessing the effects of a combination of lenalidomide and alpha-galactosylceramide-loaded monocyte-derived DCs in patients with SMM showed substantial activation-induced changes in the NKT cells, as well as activation of NK cells and a decrease in M spike during therapy, suggesting potential for targeting NKT cells against MM (55).

Innate lymphoid cells (ILCs) are a relatively recently discovered innate immune cell type related to NK cells and T cells. Specifically, ILC1s, ILC2s, and ILC3s are the innate analogues of the adaptive Th1, Th2, and Th17 cells, just as NK cells serve as the innate counterpart of cytotoxic CD8+ T cells. Recently, ILCs have been suggested to have potential effects on cancer growth and immunosurveillance (56, 57). In a study analyzing both circulating and BM ILCs, patients with MGUS were found to have increased BM ILCs, but not increased circulating ILCs, relative to healthy donors. Furthermore, patients with MGUS had increased IFNγ-producing ILC1 relative to healthy donors, but asymptomatic patients with MM had a drastic decrease. These data suggest a potential role of altered ILC populations and function in the evolution of MGUS and MM (58). ILCs express high levels of some of the targets of immune-modulatory drugs, such as pomalidomide, and are activated early following the exposure to these drugs *in vivo* (58). More studies are needed to better elucidate the roles of ILCs in the progression of MM.

Various cells of the myeloid lineage, including DCs, macrophages, and myeloid-derived suppressor cells, have been observed to change over the course of the disease progression of MM. Tumor-associated macrophages (TAMs) are known to play a key role in tumor progression in a variety of cancers, with substantial pro-tumoral effects, including stimulation of angiogenesis and intravasation, promotion of tumor cell growth, and immune suppression, by diminishing the anti-tumor activity of T cells and NK cells (59, 60). Similarly, changes in the macrophage population in the BM microenvironment have been observed over the course of the disease progression of MM (60). M2 macrophages are significantly increased in the BM of patients with MM relative to patients with MGUS, patients with SMM, and healthy donors; this effect appears to be largely mediated by increased levels of CXCL12 and IL-10 in the tumor microenvironment. These macrophages allowed for tumor proliferation as well as the suppression of the proliferation of T cells, linking changes in both innate and adaptive immunity over disease progression (61). The BM macrophages in MM have been observed to be vasculogenic, an ability absent in TAMs of MGUS (62). This finding aligns with the evidence that neovascularization is increased in MM relative to MGUS (63). TAMs in MM also appear to have increased IL-6 and IL-10 expression, along with decreased IL-12 and TNF-α expression, leading to tumor growth and immune suppression (64).

Myeloid-derived suppressor cells (MDSCs) and immature myeloid-lineage cells are now known to play a role in suppressing both innate and adaptive immune responses in the tumor microenvironment in many cancers. MDSCs have been observed to have changes in number, phenotype, and function among healthy donors, patients with MGUS, and patients with MM, suggesting a role in MM disease evolution (65). MDSCs can be divided into two subsets: CD15+ granulocytic MDSCs (G-MDSCs) and CD14+ monocytic MDSCs (M-MDSCs). In a study comparing the BM of healthy controls, stable MM, and progressive MM, proportions of G-MDSCs were significantly higher in both stable and progressive MM than healthy controls; furthermore, the proportion was found to be significantly higher in progressive MM than in stable MM. The increased number of MDSCs also significantly correlated with the BM regulatory T cell (Treg) population. In culture, these MDSCs were seen to induce immunosuppressive Tregs and inhibit other T-cell responses, contributing to immune suppression in MM (66). Increased levels of CD14+HLA-DR-/low MDSCs have also been observed in MM (67). Another study reported a significant accumulation of CD11b+CD14-CD33+ immunosuppressive MDSCs in the BM of newly diagnosed patients with MM. To further explore this role, S100A9 knockout mice, which are deficient in MDSC infiltration of tumors, had significantly reduced tumor MDSCs and MM cells relative to wild type, suggesting that accumulation of MDSCs plays a key role in tumor progression (68). The mechanisms underlying the increase in MDSCs need further study. Studies on the Vk*MYC model have suggested a role for niche-derived IL18 in promoting MDSC-mediated suppression as a therapeutic target in MM (69).

Dendritic cells play an important role in anti-cancer immunity, helping to recruit T cells, present tumor-associated antigens, and coordinate the immune response. Higher infiltration of active DCs into the primary tumor has been seen to correlate with increased patient survival in many solid tumors. However, tumors have also been recognized to escape the immune system by inducing DC dysfunction or apoptosis. Several studies have shown that MM lesions are commonly infiltrated with DCs, although the earlier findings reported that tumor-infiltrating DCs infected with Kaposi-sarcoma herpes virus were not reproduced (70). DCs likely play a multifaceted role in MM biology with both immune as well as non-immune implications. DCs can be divided into two main categories, namely myeloid or conventional DCs (mDCs) and plasmacytoid DCs (pDCs), with mDCs playing a more antigen-presentation role and pDCs secreting Type I interferons (71, 72). Both subsets have been observed to have altered distributions in MM. One study found that both mDCs and pDCs accumulate in the BM over the progression from MGUS to MM. When stimulated with apoptotic tumor cells, mDCs and pDCs from the BM of both patients with MGUS and MM exhibited increased production of IL-12 and IFN-α, respectively (70). DCs have been shown to activate Tregs as well as Th17 cells in MM (73, 74) and promote tolerance to tumor antigens and T-cell evasion *via* interactions of CD80/CD86 with CD28 on tumor cells (70). However, DCs can also directly promote the growth and survival of tumor cells and may also impact genomic instability in MM cells (20, 75, 76). In addition, DCs have also been implicated as precursors for the formation of osteoclasts in the tumor microenvironment (77–79). Together, these studies suggest that DCs may impact both immune as well as non-immune aspects of tumor biology.

Collectively, it appears that alterations in innate immunity occur early in myelomagenesis, with early alterations in NK cells, NKT cells, and ILCs, which are evident in MGUS as well as myeloma. Changes in innate immune function and populations may contribute to the progression from MGUS to MM, as NK cells appear to lose cytotoxic function, pro-tumor M2 macrophages accumulate, and MDSCs and DCs increase immunosuppressive activity. Furthermore, observed changes in myeloid lineage cells may play a role in other immune changes observed in MM, as they directly contribute to the substantial changes in adaptive immunity seen over the course of the disease as well.

### Alterations in Adaptive Immunity

#### T Cells

T cells play a key role in anti-tumor immunity. Substantial quantitative, phenotypic, and functional changes in the population of T cells have been observed over the evolution of MM. Overall, increased proportions of memory T cells and depletion of naïve counterparts have been observed in MM and MGUS, relative to healthy controls (44). Clonal CD8+ T cell expansions were noted significantly more frequently in patients with low tumor burden (MGUS or early-stage MM) compared with those with advanced disease (30). T cells from the BM of MGUS have been observed to mount a tumor-specific response to malignant plasma cells; however, T cells from MM lacked tumor-specific effector function, suggesting a role for the loss of anti-tumor T cell function in disease progression (34). Despite this finding, these MM T cells still demonstrated the ability to mount an anti-tumor cytolytic response when activated by tumor-loaded DCs, indicating that even in advanced MM, T cells can be recruited to mediate anti-MM activity (80). The capacity of the endogenous T cells to mediate anti-tumor function *in vivo* has now been translated to clinical studies with the efficacy of bispecific antibodies (81).

Persistent antigen stimulation in the setting of cancer leads to the emergence of T cell exhaustion (82). CD8+ T cells in MM have been observed to have features associated with senescence and exhaustion, expressing proteins such as PD-1, CD160, CTLA-4, and CD57 (29, 83–85). Interestingly, recent data suggest that the features of T-cell exhaustion begin early and may manifest as early as MGUS (42). As the MGUS phase lasts for several decades, this raises a question as to how exhausted clones can be maintained over prolonged periods of time. A possible solution from murine models is the appreciation that chronic persistence of immunologic memory depends in part on the presence of a subset of stem-like memory T cells, which are marked by the expression of a transcription factor TCF-1 (82). Indeed, recent studies have suggested that transition from MGUS to MM is associated with the attrition of TCF1hi stem-like memory T cells, as well as T cells expressing markers associated with tissue residence (86), and instead accumulation of senescent T cells expressing high levels of lytic genes and senescent markers (42). These cells exhibit decreased proliferation and impaired cytotoxic function and fail to produce IFN-γ when stimulated (38). The senescent phenotype described in MM cells includes a distinct telomerase-independent phenotype, which may not be reversible (84). However, data from studies on T-cell redirection does support the capacity of endogenous T cells to mediate anti-tumor function *in vivo* (81).

Oligoclonal expansions of CD8+ CD57+ terminal effector T cells (T_TE_ cells) have been observed in the BM and the peripheral blood of patients with MM (87, 88). Treatment with thalidomide led to an increased expansion of cytotoxic T-cell clones, and these expansions correlated with increased progression-free survival (32). The T_TE_ cells have relatively low PD-1 expression, making the cell subset a less appealing target for PD-1/PD-L1 blockade (89). CD69 may be a marker for T-cell activation or tissue residence (86). Further, the division of these cells by CD69 expression has shown altered proportions of BM C69+ and CD69-T_TE_ cells among controls, MGUS, SMM, and MM. The CD69- cells showed oligoclonal expansions capable of lysing autologous tumor cells, while the CD69+ cells showed an increased inhibitory-immune checkpoint expression (88).

An important feature of exhausted T cells is the expression of inhibitory checkpoints, such as PD-1 (85, 90). In a murine model, MM-specific T cells were shown to express higher levels of PD-1 when compared to non-reactive T cells (91). The expression of PD-L1 on MM plasma cells has also been observed to be higher than that of MGUS and healthy plasma cells (92, 93). Increased PD-L1 expression has also been described in persistent minimum residual disease (MRD+), after treatment (94). The PD-L1 expression also was observed to correlate with an increased risk of progression of SMM (29). Secretion of IL-6 by BM stromal cells induced PD-L1 expression on MM cells *via* signaling through JAK2, STAT3, and MEK1/2 (93). Activation of the JAK/STAT pathway by IFNγ secreted by the BM immune cells as well as toll-like receptor (TLR) stimulation may also contribute to MM cell PD-L1 expression (92, 95). Another analysis of BM from patients with MM, SMM, and MGUS revealed a correlation between increased T-cell exhaustion and senescence and disease progression, particularly in tumors with trisomies (96). These considerations have led to studies targeting the PD1 and PD-L1 pathway in clinical MM as well as SMM (90, 97). The clinical results from these studies, however, have been underwhelming to date. Recent data also suggest that other inhibitory checkpoints, such as TIGIT or Lag-3, may be important targets in MM (98), and studies targeting these pathways are currently ongoing. Recent studies have also demonstrated a role for adenosine signaling in inhibiting the T-cell function in MM (99), indicating another potential target for anti-MM immunotherapy.

Two other aspects of T-cell function relevant to MM include regulation and altered T-cell polarization. Tregs have been extensively observed in MM. Treg populations have been observed to be increased both in MM and MGUS relative to healthy controls and were predicted to play a role in immune dysfunction observed in MM (100). A separate study observed an increase in CD4+CD25+ T cells in MGUS and MM, but a decrease in FOXP3+ Treg cells; these Tregs also appeared dysfunctional with a decreased ability to suppress T-cell proliferation, potentially contributing to a non-specific increase in T cells and dysfunctional anti-tumor immunity (101). Mechanisms underlying the increase in Tregs involve both direct induction by tumor cells, as well as *via* cross-presenting DCs (73, 102). Mice lacking Tregs, achieved *via* knockout of FOXP3, had prolonged survival after injection of Vk*MYC MM cells compared to wild-type mice, further emphasizing the role of Tregs in immune suppression and MM progression (103). Together, these studies suggest a role for Treg-mediated regulation in MM immunity. Immunotherapy targeting Tregs has shown promise for certain types of cancer in preclinical models (104); as these methods evolve, they may eventually have a role in MM treatment.

Another feature of MM-associated T cells is an increase in IL-17 producing T cells (74). Th17 cells have been observed to be increased in frequency in the BM of patients with MM compared to patients with MGUS and healthy donors (74, 105). The IL-17 secretion by these Th17 cells increases osteoclastogenesis and activation and contributes to MM bone disease (106). The Th17 IL-17 secretion was also observed to promote MM cell growth *via* its receptor. Furthermore, these Th17 cells significantly inhibit the production of pro-inflammatory Th1 cytokines such as IFN-γ, suggesting a role in maintaining immune suppression in the tumor microenvironment (105, 107). In Vk^*^MYC mice, increased Th17 cells favored progression of MM, and treatments blocking IL-17 delayed disease progression (108). Another study in the Vk^*^MYC mouse MM model further suggests that T cells are a key controlling agent in MM and again implicates IL-17 in disease progression. With autologous BM transplant in these mice, robust tumor control was observed in mice with distinct TCR repertoires. Generation of MM-specific T cells was also observed after transplant. Furthermore, IL-17 was observed to promote tumor growth and MM relapse, while IFN-γ secretion appeared critical to anti-tumor responses (109). From these data, it appears that therapies targeting IL-17 secretion by Th17 cells may have a positive impact both on MM bone disease and tumor control.

Immune properties of long-term survivors in MM may provide further insight into potentially key immune populations for sustained tumor control. Comparison of peripheral blood immune cells between long-term survivors (those surviving >10 years) and patients with <10 years follow-up revealed significantly higher frequencies of clonal cytotoxic T-cell expansion in the long-term survivors. Furthermore, long-term survivors had higher circulating Th17 cells and lower Tregs compared to other patients with MM (110). Circulating immune cell populations after autologous stem cell transplant also correlate with patient survival. Namely, increased circulating lymphocytes, decreased circulating monocytes, and an increased lymphocyte to monocyte ratio all significantly predicted improved treatment-free survival, further suggesting a role for lymphocyte immune surveillance against MM and delayed disease progression in patients with positive responses (111).

These data together show that there are major changes in T-cell states associated with MM progression. However, the degree to which these changes reflect cause vs. effect of the underlying disease progression remains to be established. Features of T-cell exhaustion appear as early as MGUS, but MM is associated with the attrition of stem-like memory T cells and instead accumulation of effector cells expressing lytic genes.

#### B Cells

As MM is a tumor of B-cell lineage, these cells have also been extensively studied in MM, and the most notable feature of MM is the depletion of normal immune globulins. While the contribution of B-cell lineage to the clonal compartment and its evolution is outside the scope of this review, we will briefly discuss the potential contributions of regulatory B cells (Bregs) in tumor immunity. Bregs have been proposed to play a role in immune modulation, suppression, and tolerance. Recently, these cells have been implicated in the maintenance of immunosuppressive tumor microenvironments and impairment of T-cell mediated tumor killing (112). Interest in Bregs has increased after the appreciation that they express high levels of CD38 and may be targets of immune effects of anti-CD38 antibodies. A recent study described higher proportions of Bregs in the BM of newly diagnosed patients with MM compared to those on maintenance therapy. Furthermore, NK cells co-cultured with Bregs derived from the BM of patients showed decreased lysis of MM cells, suggesting a role for Bregs in MM immune evasion (113). While these initial studies are of interest and indicate a role for B cells in MM disease evolution, substantial further investigation will be necessary to better understand their role.

### Role of BM Niche and Bone Cells

A characteristic feature of MM is the growth of tumor cells in the BM. Therefore, MM lesions evolve in close proximity to bone cells, and MM-associated immune cells are continually modified by signals derived from the marrow. Co-culture of MM cells with T cells induced RANKL expression and secretion by the T cells, caused in part by MM cell secretion of IL-7 (114). In addition to overactivation of osteoclasts, MM bone disease also has contributions from osteoblast inhibition (115). Co-culture of MM cells with osteoprogenitor cells inhibited differentiation of mature osteoblasts; blockade of Runx2/Cbfa1 activity was the observed mechanism. This blockade was mediated by cell-to-cell contact and interaction between VCAM-1 on osteoprogenitors and VLA-4 on MM cells. Furthermore, BM biopsies of patients with MM revealed significantly fewer Runx2/Cbfa1-positive cells in patients with osteolytic lesions (116). IL-3 secretion also appears to play a role in osteoblast inhibition, osteoclast stimulation, and MM bone disease. IL-3 levels in the BM of MM have been observed to be increased relative to both healthy controls and in patients with MGUS; this IL-3 contributed both to bone destruction and MM cell growth (117). The IL-3 in the BM microenvironment is also derived from T cells—MM T cells can express IL-3 (118). Furthermore, the IL-3-dependent effects appeared to be mediated by monocytes and macrophages, further emphasizing the complex interactions between tumor, bone, and immune cells over the evolution of MM bone disease (119). This data also suggests potential for the modulation of IL-3 as a target to combat MM bone disease.

Dikkopf 1 (DKK1), a Wnt inhibitor, has been observed to be overexpressed in malignant plasma cells in patients with MM who have focal bone lesions. DKK1 expression has also been observed to be significantly increased in MM plasma cells relative to both MGUS and healthy controls, and BM DKK1 levels are a risk factor for the progression of SMM (120–122). DKK1 directly inhibits osteoblast differentiation *via* Wnt inhibition (123). Furthermore, DKK1 increases osteoclast function *via* disruption of osteoprotegrin and RANKL expression by osteoblasts (124). In addition to contributing to MM bone disease, DKK1 has recently been implicated in altering T cells in the BM of MM. TCF1, a marker of stem-like memory T cells, is known to be regulated by Wnt signaling. Patients with MM who have elevated DKK1 levels were observed to have reduced levels of TCF1+ memory T cells, suggesting a role of DKK1 from MM cells in depleting stem-like memory T cells (42).

Osteoclasts with increased function due to interactions with MM cells go on to alter the BM environment in ways conducive to further MM expansion and immune suppression (125). They can enhance the activity of Treg cells *via* antigen presentation and secretion of IL-10 and TGF-β (126, 127). Osteoclasts can also directly inhibit T-cell cytotoxicity and anti-tumor activity *via* the expression of immune checkpoint molecules, including PD-L1, CD200, herpes virus entry mediator, and Galectin-9. These osteoclasts also express T-cell metabolism regulators, such as IDO and CD38 (128). As these molecules contribute to MM immune evasion, they represent potential therapeutic targets, some of which are already being explored. Therefore, in addition to immune dysfunction and suppression induced over disease progression by direct interactions between MM cells and immune cells, indirect immune modulation by MM cells *vi*a osteoclast activation appears to play a significant role in the coevolution of tumor and immune changes.

IL-6 is known to play a central role in encouraging survival and proliferation of malignant plasma cells *via* its receptor, IL-6R. This IL-6 can also induce the expression of PD-L1 on MM cells, aiding in immune evasion (93). The main source of BM IL-6 is BM stromal cells. Additionally, some MM cells can produce their own IL-6, and macrophages, osteoblasts, and osteoclasts also contribute to BM IL-6 levels (129, 130). Serum IL-6 has been observed to be increased in MM relative to MGUS, as well as in patients with advanced MM compared to patients with early or plateau-phase disease. Furthermore, elevated IL-6 levels have been observed to correlate with higher disease activity and poor prognosis (131). Additionally, increased soluble IL-6R levels in patients with MM have been associated with shorter survival, further implicating interactions between IL-6 and IL-6R in disease progression (132, 133). Therefore, strategies targeting both IL-6 and IL-6R are being explored. Direct targeting of IL-6 *via* monoclonal antibodies has shown promise *in vitro* and in mouse models against MM cell lines (130). Strategies to downregulate IL-6R on MM cells are also being explored, including metformin treatment and pharmacologic inhibition of histone deacetylase 3, both of which have been shown to decrease MM proliferation *in vitro* (134, 135). A recent clinical trial of siltuximab, a monoclonal antibody targeting IL-6, in SMM failed to meet the pre-specified endpoint criteria but did show promise for delaying high-risk SMM progression (136). While these approaches have not yet had a large clinical impact on MM, the IL-6 – IL-6R axis remains an interesting target for MM therapy and prevention of disease progression.

## Evolution of the Tumor-Immune Interface

**Figure 1:**
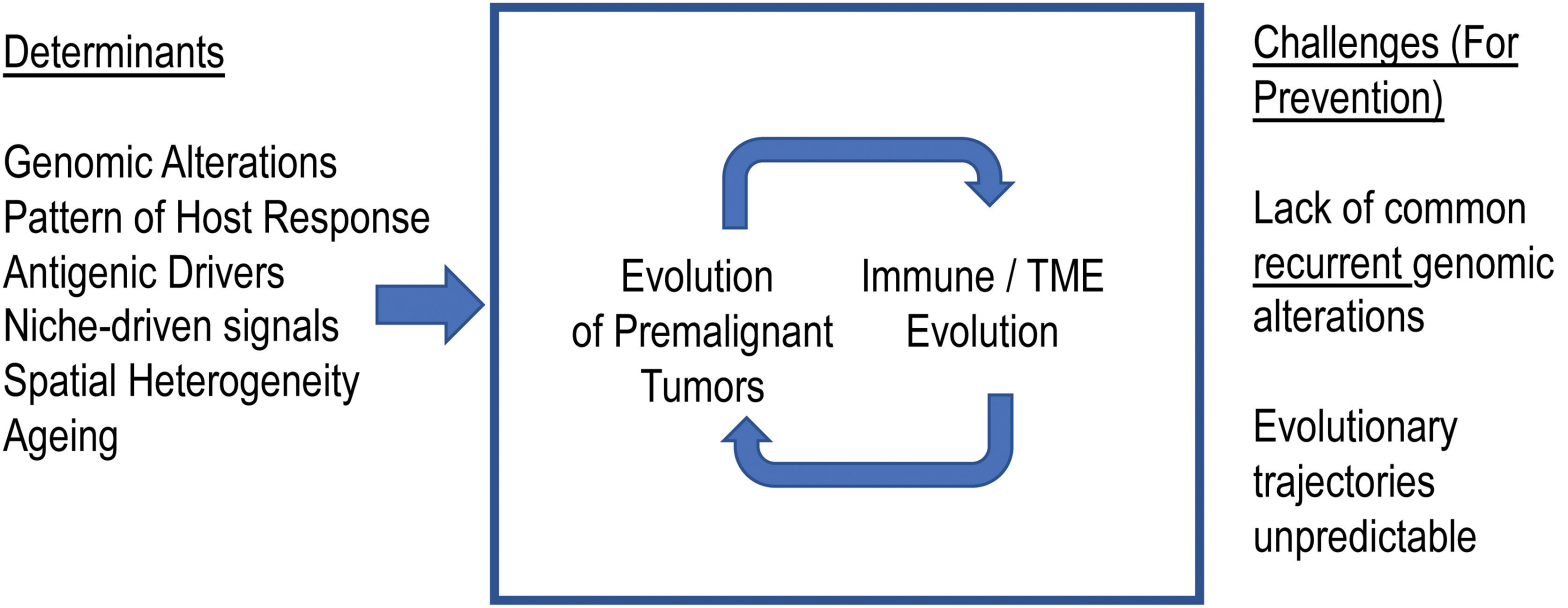
Co-evolution of myeloma precursor states and immune microenvironment. We propose that the malignant transformation of the precursor states to myeloma depends not just on evolution of tumors but also on the immune microenvironment. Understanding the principles underlying these evolutionary trajectories will enable effective immune prevention.

The studies discussed above paint a complex picture with multifaceted interactions between MM cells and immune cells. These interactions include signals that both promote as well as suppress tumor growth. However, neither tumors nor the immune system are static entities. Both represent highly adaptive and complex biologic systems that have the capacity to evolve over time. In the case of MM, which is a tumor of an immune cell (plasma cell), both systems may also share a common niche in the BM. Therefore, in terms of evolutionary principles for the interaction of species, the interactions may involve competition as well as immune predation. Therefore, we suggest that this biology will depend both on tumor intrinsic as well as extrinsic elements (Figure 1). Some other elements that uniquely apply to MM include the presence of underlying antigenic triggers and interactions with bone cells.

### Tumor Intrinsic Factors: Genetics, Instability, Proliferation, and Antigenic Triggers

As discussed earlier, MM tumors are highly heterogenous and vary in terms of underlying oncogenic drivers, degree of genomic instability, cell cycle checkpoints, and proliferation rate. While these considerations often form the basis of clinical “risk phenotypes,” they are also likely to impact the fitness of the tumors in terms of being able to compete for resources, such as growth factors and nutrients, and either overcoming or escaping the effect of immune predation. It is therefore essential that the next generation of studies understanding the immune biology of MM is carried out in the context of the underlying genetics of the tumors. MM and MGUS are more common in the black population, and a deeper understanding of the mechanisms underlying race-dependent differences in the risk of transformation of precursor states is needed, particularly as these patients have been underrepresented in the existing studies on MM biology and therapy (137). Recent studies on other hematologic malignancies, such as leukemia, find a correlation between clinical/genomic risk and immune phenotypes (138), but such studies on MM or MGUS are lacking. One of the challenges in MM research is that the current mouse models do not recapitulate the oncogenic drivers found in human MM and do not recapitulate its genetic diversity (139). Therefore, there remains an unmet need to better understand the changes in preneoplastic cells, altered immune surveillance, and the nature of MGUS-specific immune responses that contribute to this process.

It is also increasingly appreciated that MM is likely to originate in the context of a polyclonal immune response (5). This is supported both by recent genome sequencing studies (3) as well as *in vivo* modeling of disease states, such as Gaucher disease (140, 141), wherein the risk of MM is increased. Interestingly, in settings, such as Gaucher disease, wherein the underlying antigen driving the gammopathy is known, tumor cells remain responsive to underlying antigenic triggers *in vivo* in preclinical models (141) and respond to targeting the underlying antigen in patients (142). Targeting the underlying antigenic trigger may, therefore, potentially change the evolutionary trajectories of these precursor lesions.

An important component of tumor-intrinsic features that may impact immune surveillance may be their recognition by antigen-specific immune cells. Malignant plasma cells in MM have long been known to exhibit a capacity for antigen presentation (143). These cells also express many molecules involved in interactions with immune cells in the BM microenvironment. Both MGUS and MM malignant plasma cells have been observed to have an increased expression of CD86. Overall, these data suggest increased antigen presentation/costimulation in MGUS that decreases over progression to MM (144). Alterations in the antigen-processing machinery (APM) of plasma cells have been observed in MGUS and MM, and this may contribute to the evasion of immunosurveillance; additionally, the progression of MGUS was correlated with the expression of APM-related factors, such as calnexin and calreticulin (145). Furthermore, MM plasma cells have been observed to have increased PD-L1 expression relative to MGUS, further connecting tumor evolution to immune alterations (93, 95).

### Immune Intrinsic Features: Persistence, Function, and Spatial Aspects

As with tumor genetics, the immune microenvironment in each patient with MM or MGUS is distinct. This has become particularly evident in the recent application of high-content single-cell approaches to study these tumors (42). As the evidence of immune activation and exhaustion appears early during the pathogenesis of MGUS (42), the features of the immune response that are essential to maintain long-term stability and persistence of immunity, such as the presence of stem-like memory T cells, are likely to be the critical determinants of immune control. Specific targets of the immune response may also be critical, as it may be more important to target clonal, as opposed to subclonal, alterations or to target critical properties of the clone, such as stemness. Finally, although spatial aspects of the immune response in hematologic malignancies have been understudied, it has been well-known to pathologists and radiologists that myeloma grows in a multifocal pattern, hence the name multiple myeloma. Understanding the spatial aspects of the immune response as well as tissue resident vs. recruited cells may therefore be a critical element of immune control (86).

### Evolutionary Trajectories

Evolutionary features of tumors can be understood based on the balance of evolutionary and ecologic diversity (146). Evolutionary diversity is represented by tumor heterogeneity in terms of genomic makeup as well as changes in this over time. Ecologic diversity is impacted by hazards, such as immune cells, and the availability of resources, such as growth factors and nutrients (146). In the setting of MGUS, much of the evolutionary diversity is established early but changes over time can vary. These considerations, however, also suggest that a critical determinant of the evolutionary trajectory may be related to ecologic features, including the hazards posed by the immune system. However, as these interactions are likely to be regional, the attention to spatial aspects of these interactions will be essential to better understand critical regulators of tumor evolution.

## Implications for Immune-Prevention

With the expanding evidence of immune changes accompanying the progression from MGUS to SMM to MM, the next step is to leverage this knowledge to delay or even prevent disease progression. The standard of care for both MGUS and SMM has traditionally been observation (147, 148). However, recent clinical trials now seek to leverage the immune system to delay the presentation of symptomatic MM. Early trials investigating the treatment of SMM with melphalan and prednisone, thalidomide, thalidomide plus pamidronate, and other combinations did not lead to the improvement in patient outcomes relative to observation, although some increase in initial responses to treatment were seen (149–151). A subsequent phase 3 randomized trial stratified by risk revealed that high-risk patients with SMM, identified by biomarkers such as high M-protein levels, benefited from lenalidomide plus dexamethasone, with significantly increased time to progression and overall survival compared to observation (152). In a recent randomized trial comparing lenalidomide to observation in SMM, the lenalidomide group showed a significantly longer time to progression to symptomatic MM, providing promising early evidence for immunomodulatory methods of delaying the usual disease course (153). While these studies are expected to change the current practice in a subset of patients, we need a deeper understanding of the mechanisms of response and resistance to these therapies. As discussed earlier, it is likely that different precursor lesions may follow different trajectories based on both the genetics of these lesions as well the host response. Therefore, it is unlikely that the one-size-fits-all approach will suffice for the targeted prevention of MM (154). For some lesions, such as those with a higher rate of genomic evolution, it may be essential to pursue combinatorial approaches. For others, more conservative and sequential approaches may suffice. The concept that the hierarchy of immune exhaustion begins early also suggests the need to link immune-based prevention to early detection (155) to fully realize the potential of this strategy.

## Author Contributions

All authors contributed to writing of the paper and approved the final manuscript.

## Conflict of Interest

The authors declare that the research was conducted in the absence of any commercial or financial relationships that could be construed as a potential conflict of interest.
